# Supplementary figures and images for: High throughput sequencing-based analysis of the soil bacterial community structure and functions of Tamarix shrubs in the lower reaches of the Tarim River
Source: PeerJ. 2021 Sep 8;9:e12105. doi: 10.7717/peerj.12105 (PMC8434807; doi:10.7717/peerj.12105)

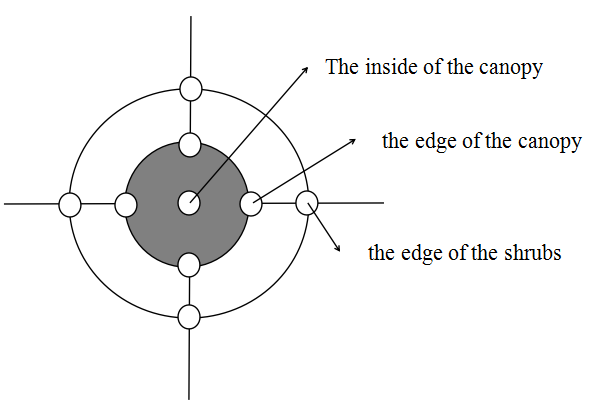

Supplement: Supplemental Information 7 [file peerj-09-12105-s007.png]
